# Supplementary figures and images for: The receptor binding properties of H5Ny influenza A viruses have evolved to bind to avian-type mucin-like O-glycans
Source: PLoS Pathog. 2026 Jan 20;22(1):e1013812. doi: 10.1371/journal.ppat.1013812 (PMC12904578; doi:10.1371/journal.ppat.1013812)

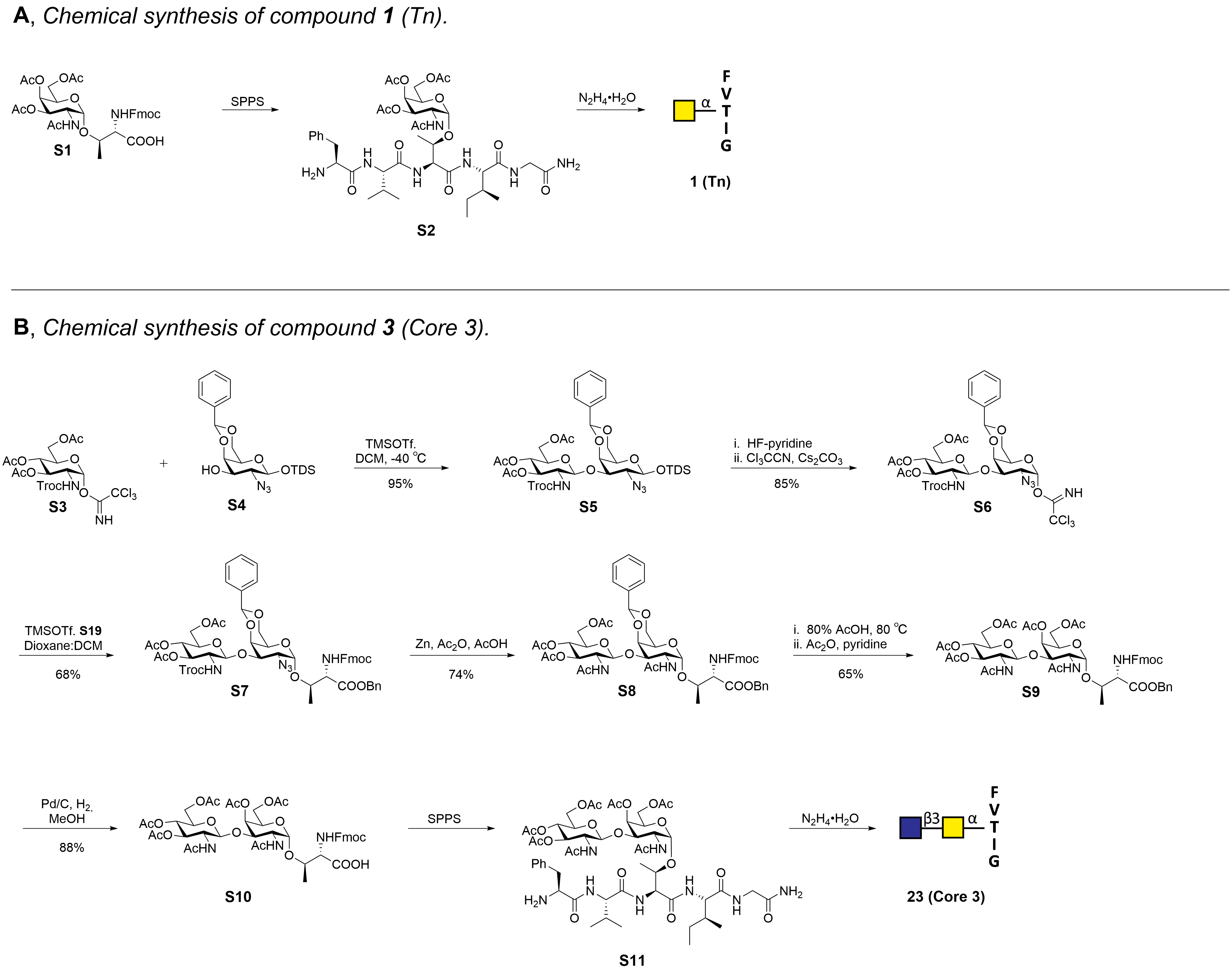

Supplement: S1 Scheme — (TIF) [file ppat.1013812.s001.tif]

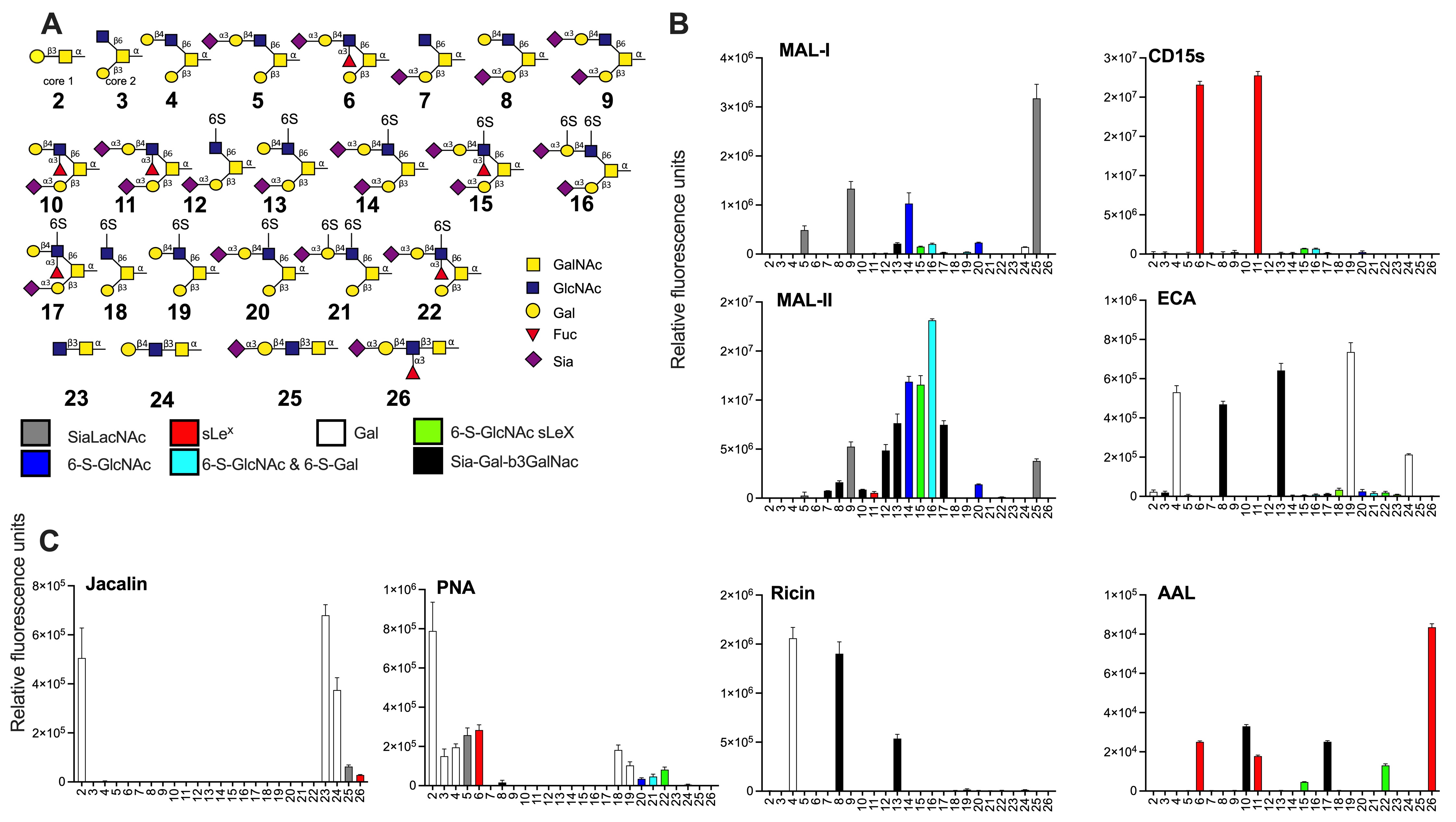

Supplement: S1 Fig — A) The O-glycans were printed on a glycan microarray, and the terminal epitopes are indicated in different colors. (B) O-glycan binding of plant lectins MAL-I, MAL-II (both binding α2,3 linked SIA), ECA (binding terminal LacNAc), and a mouse IgM antibody, CD15s, clone CSLEX1 (binding SLex) (C) The binding of plant lectins Jacalin [binds to terminal T-antigen (Gal-β1,3-GalNAc)]2, PNA (peanut agglutinin, binds to binds terminal T-antigen [Gal-β1,3-GalNAc)3, Ricin (binds to terminal type 2 LacNAc)4, and AAL (Aleuria aurantia lectin, binds to fucose)5. (JPG) [file ppat.1013812.s003.jpg]

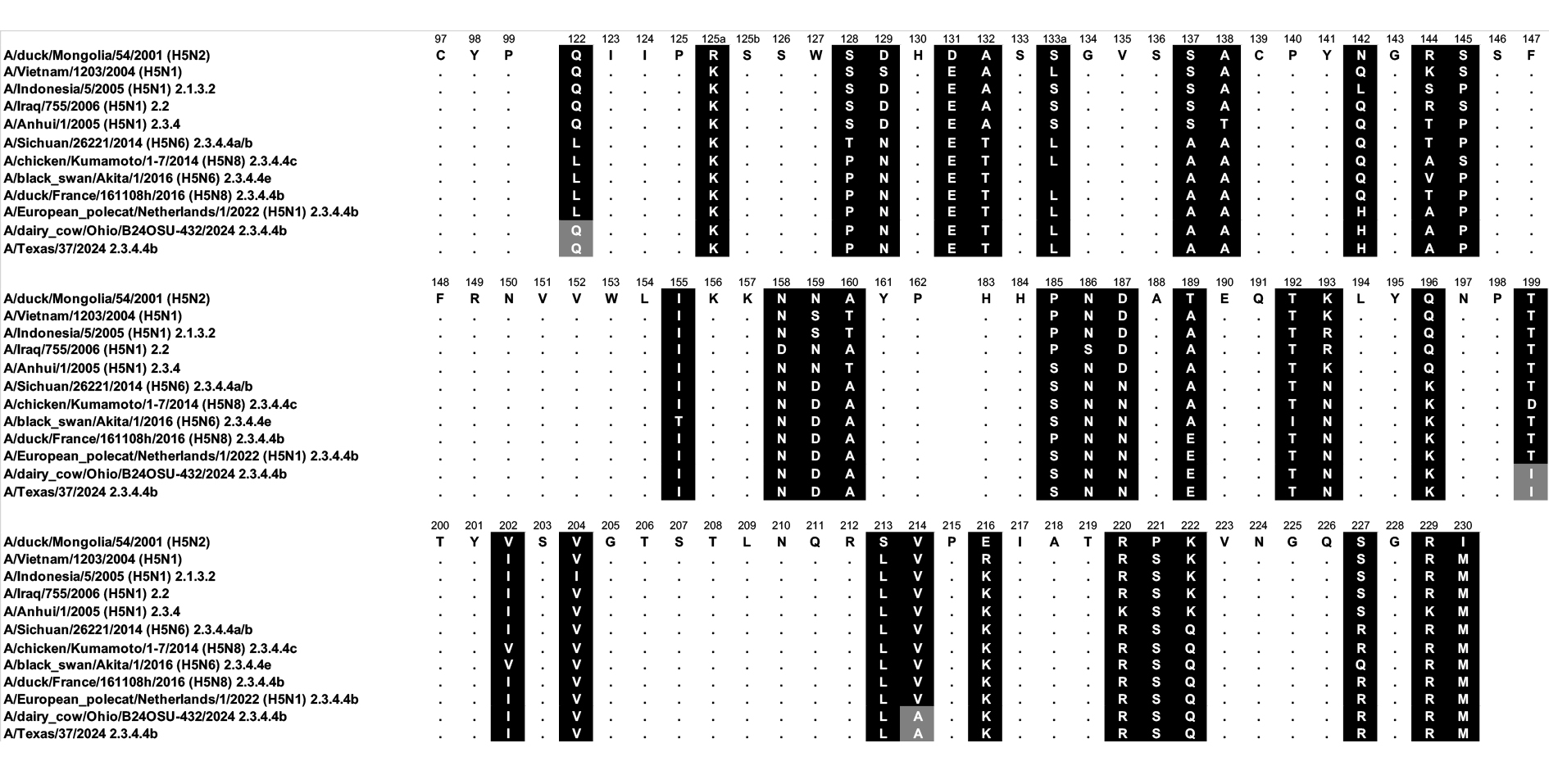

Supplement: S2 Fig — Alignment of the RBS residues with amino acid positions (H3 numbering) indicated above the alignment, non-conserved residues highlighted in a black background, dots indicating identical amino acids. 2024, dairy cattle outbreak specific amino acid changes, are highlighted with a gray background. (JPG) [file ppat.1013812.s004.jpg]

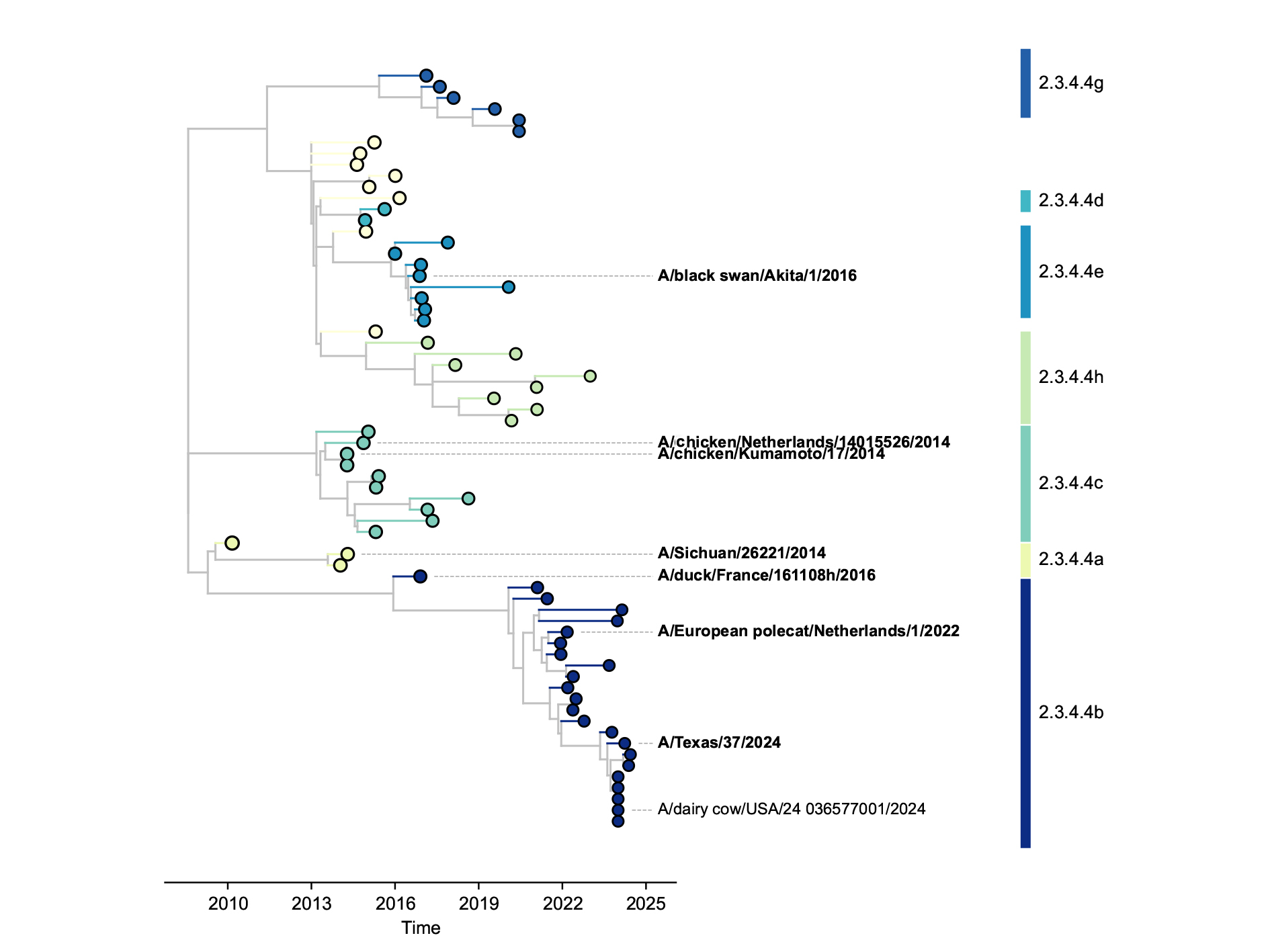

Supplement: S3 Fig — The viruses that were studied in this work are indicated. (JPG) [file ppat.1013812.s005.jpg]

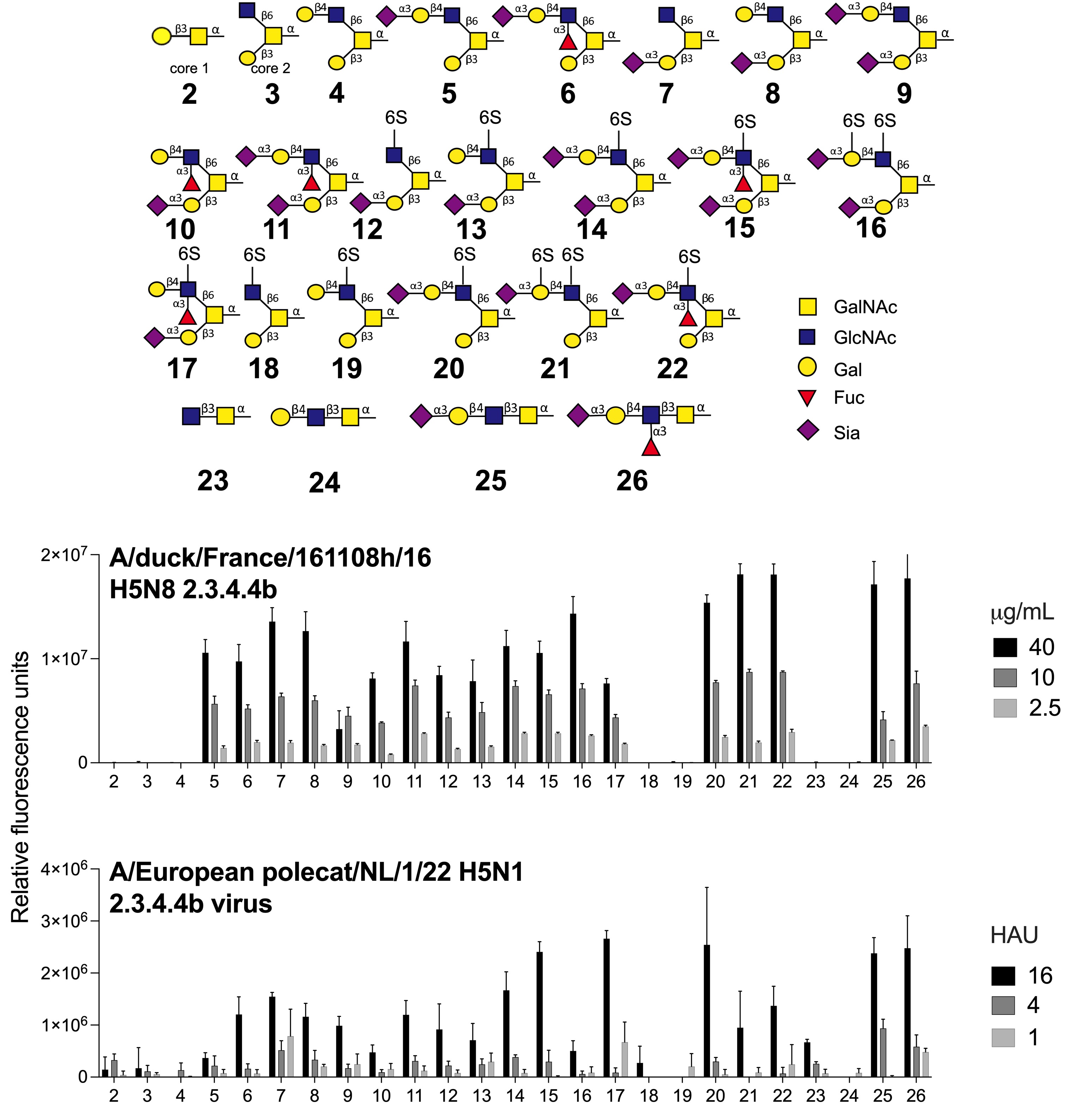

Supplement: S4 Fig — Top panel is the O-linked glycan ligands on the array and bottom panels are the binding of these ligands to both HA and virus titrated from 40 to 2.5 µg/mL and 16–1 hemagglutination units. For reference, in Fig 2, 50 µg/mL and 32 HAU units were used respectively. (JPG) [file ppat.1013812.s006.jpg]

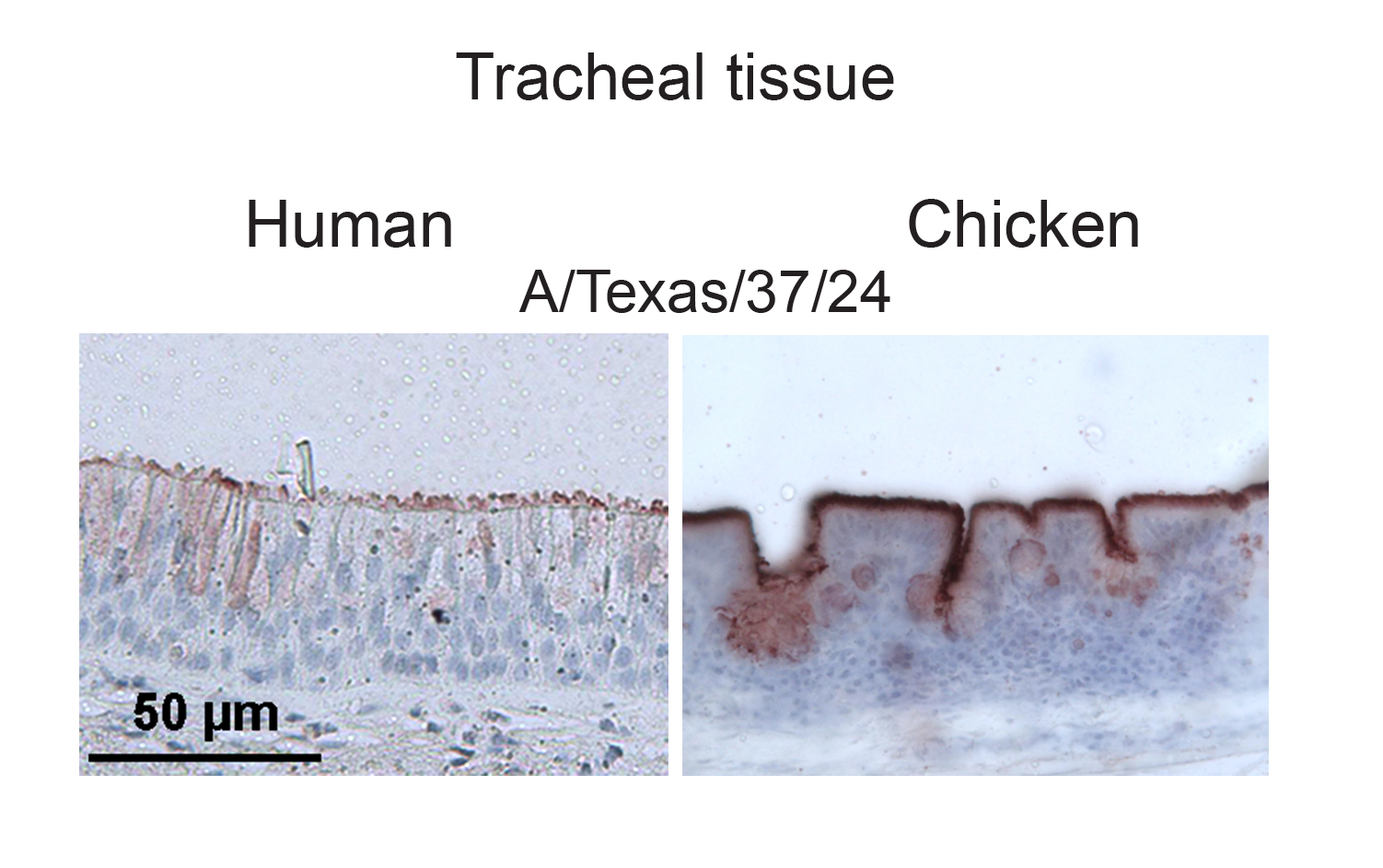

Supplement: S5 Fig — The binding to human and chicken tracheal tissue was investigated for A/Texas/37/24 H5 HA. AEC staining was used to visualize tissue binding. (JPG) [file ppat.1013812.s007.jpg]

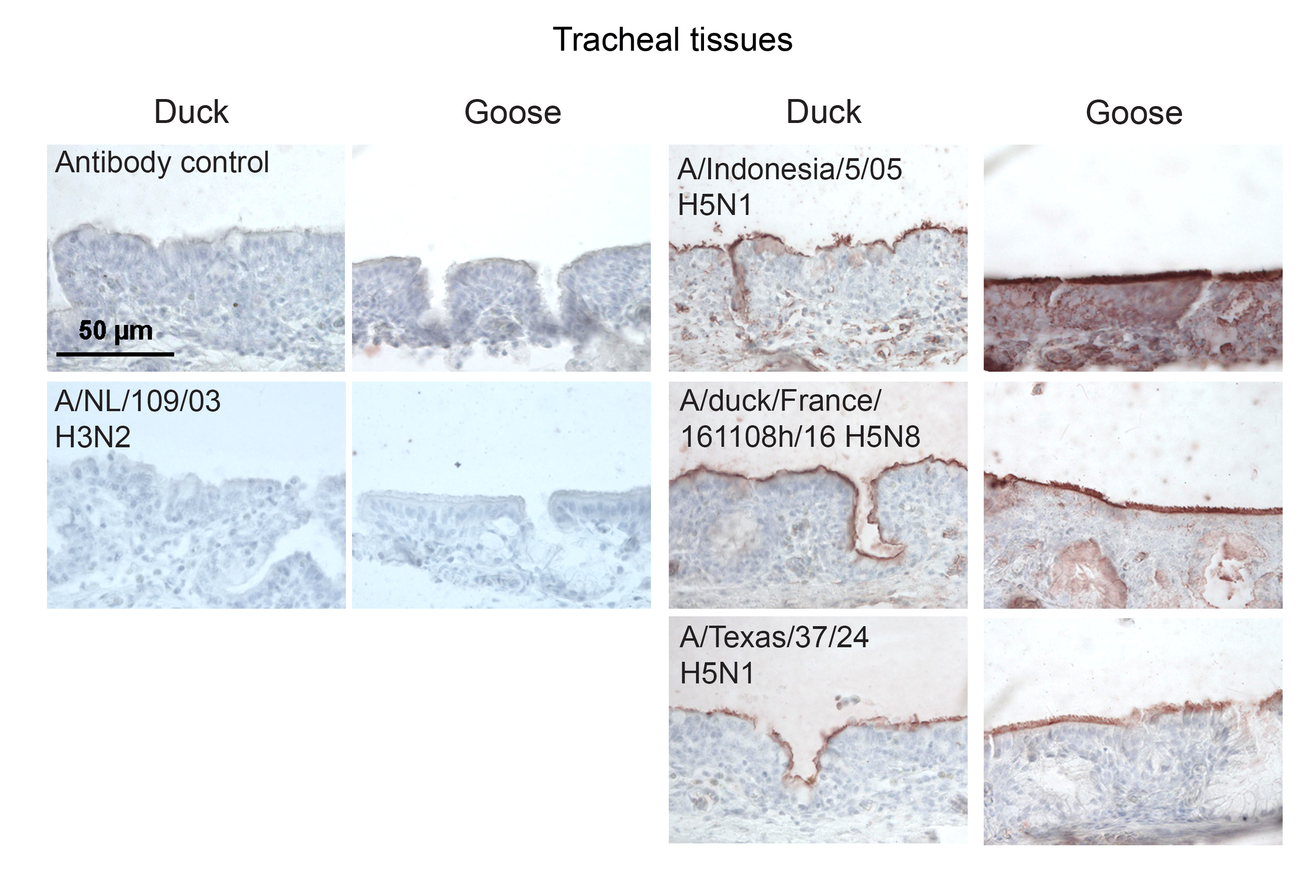

Supplement: S6 Fig — The binding to duck and goose tracheal tissue was investigated for A/NL/109/03, A/Indonesia/5/05, A/duck/France161108h/16, and A/Texas/37/24 H5 HA. AEC staining was used to visualize tissue binding. (JPG) [file ppat.1013812.s008.jpg]

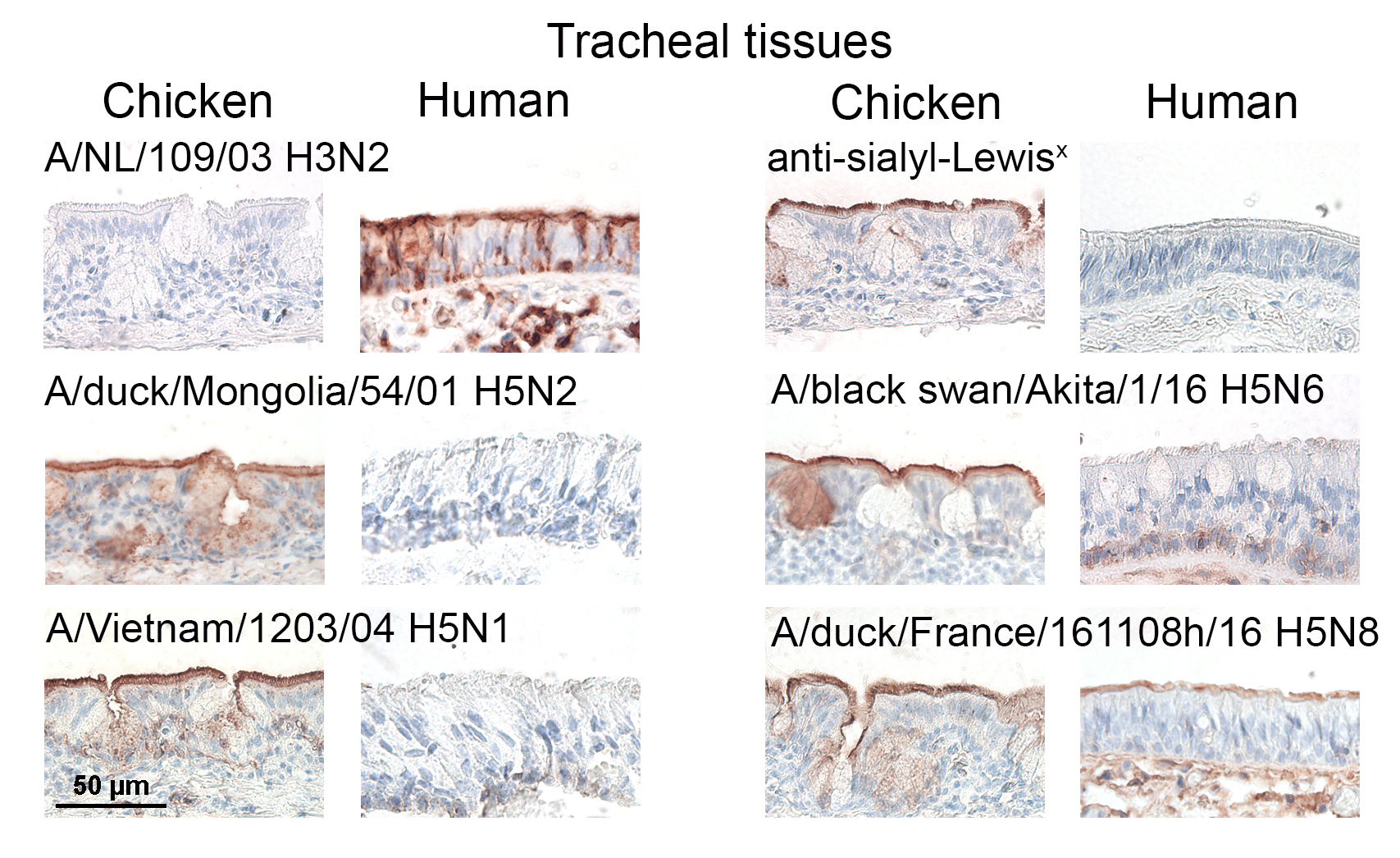

Supplement: S7 Fig — The binding to human and chicken tracheal tissue was investigated for different influenza A H5 HAs. The HA from A/NL/109/2003 was used as a positive control for human trachea binding. The antibody CD15S was used to visualize sialyl-Lewisx epitopes. AEC staining was used to visualize tissue binding (JPG) [file ppat.1013812.s009.jpg]

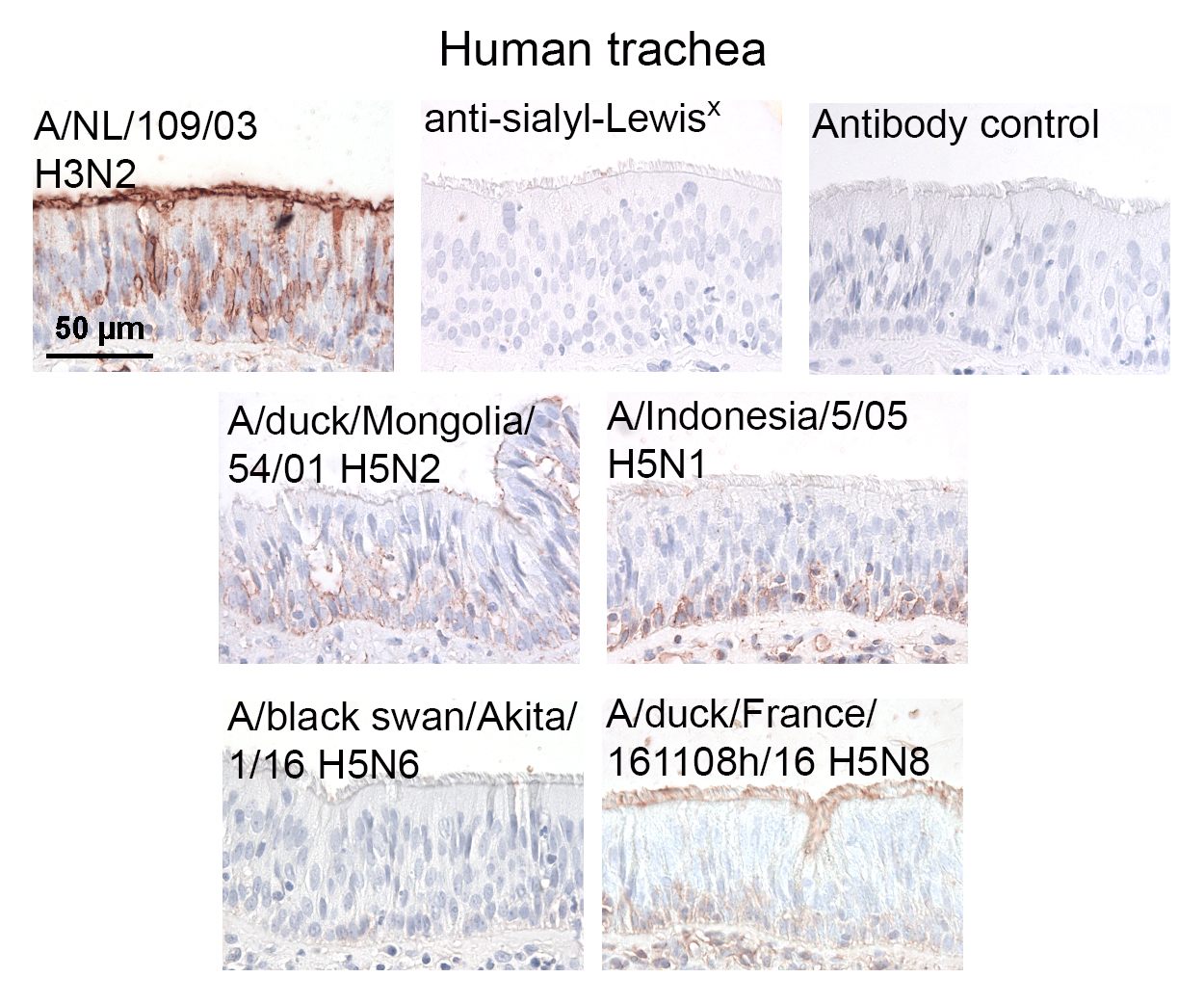

Supplement: S8 Fig — Binding experiments with several influenza H5 HAs, anti-sialyl-Lewisx antibody, and human tracheal tissues (from a different patient than in Fig 4) were performed. The H3 HA from A/NL/109/03 was used as a positive control. AEC staining was used to visualize tissue binding. (JPG) [file ppat.1013812.s010.jpg]

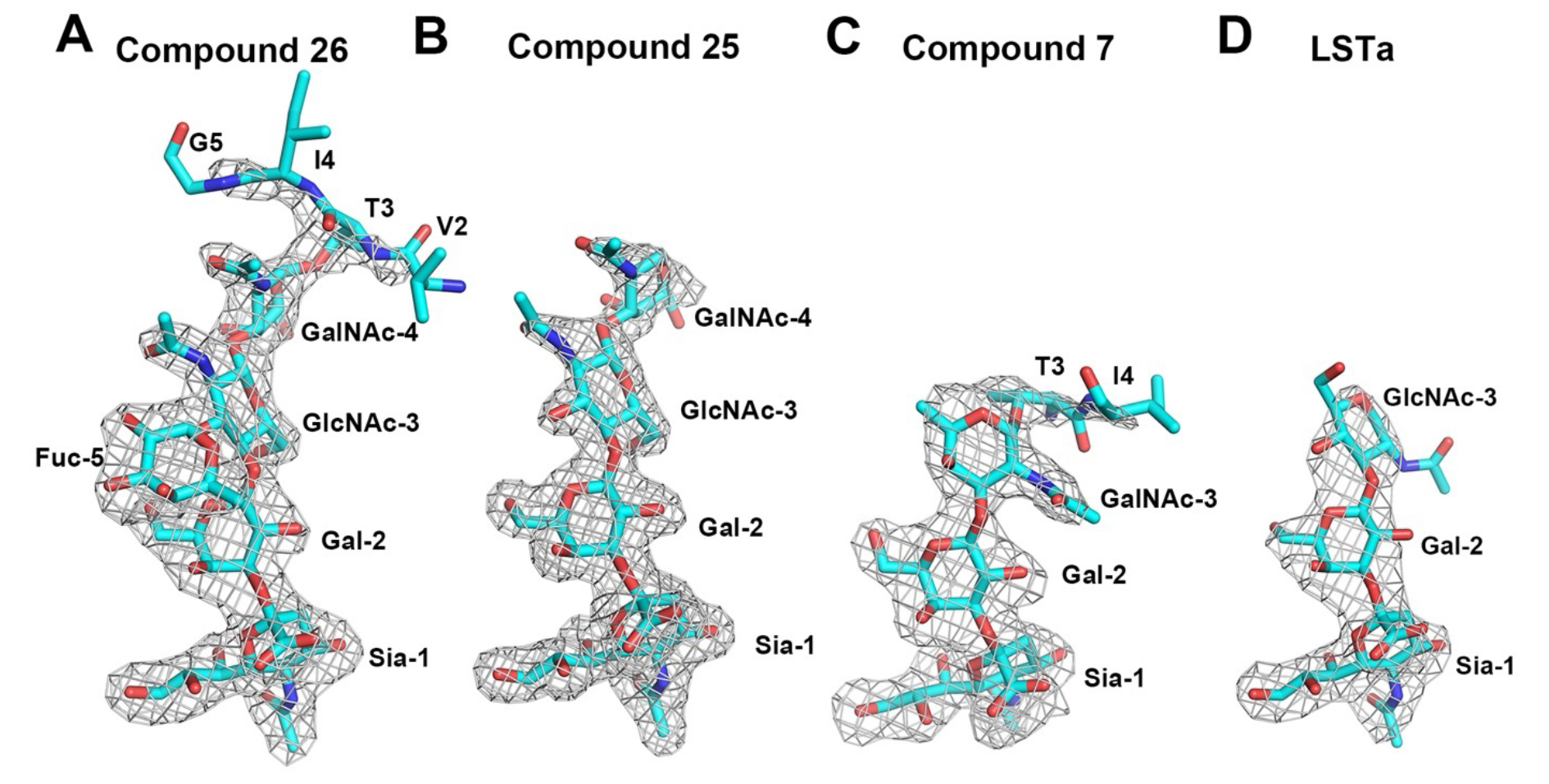

Supplement: S9 Fig — (A) Compound 26 (2.50 Å resolution). (B) Compound 25 (1.98 Å resolution). (C) Compound 7 (2.40 Å resolution). (D) Glycan LSTa (2.90 Å resolution). Some residues from the peptide linker moeity (FVTIG) of the glycan compounds (Fig 1) could also be modeled, albeit into weak electron density. The compounds are colored with cyan carbon atoms. Simulated annealing omit Fo-Fc maps are represented in grey mesh and contoured at 2.5 σ. (JPG) [file ppat.1013812.s011.jpg]

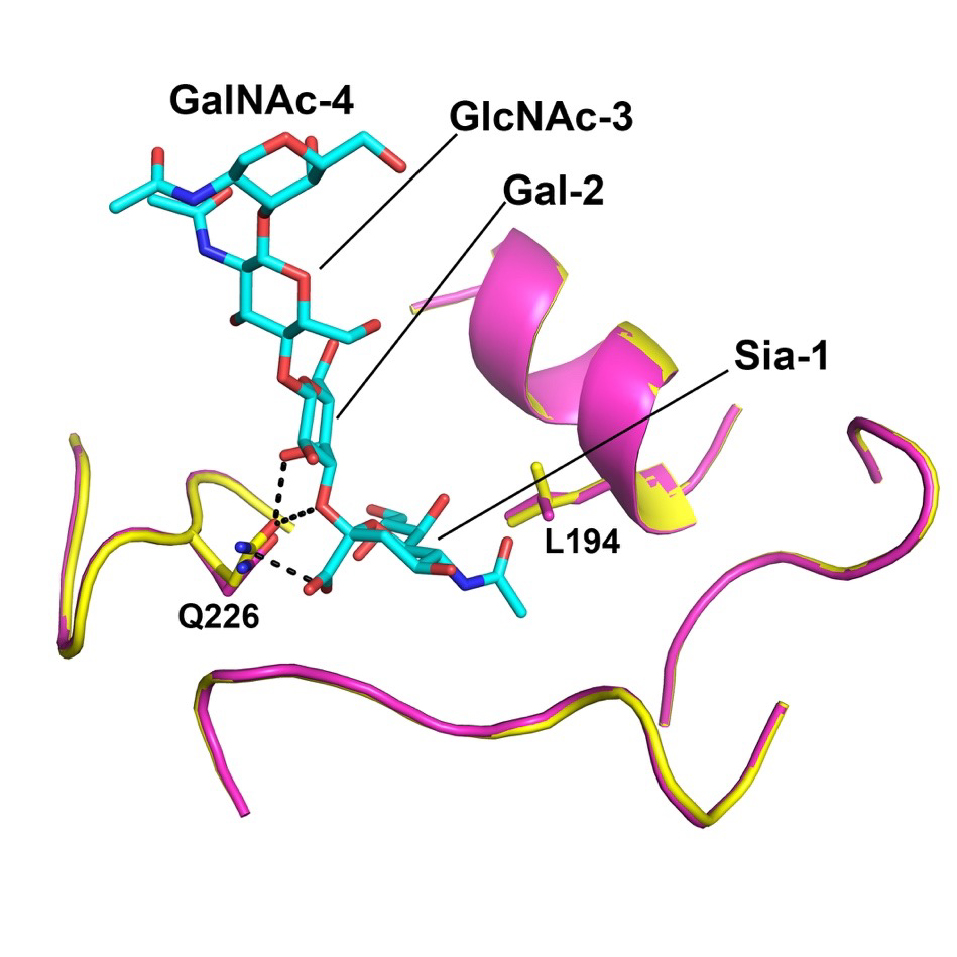

Supplement: S10 Fig — The apo HA is in pink carbon atoms, the complexed HA in yellow carbon atoms, and the ligand in cyan carbon atoms. The side chains of Q226 and L194 with very minor conformational changes are shown for comparison to the unaltered backbones. (JPG) [file ppat.1013812.s012.jpg]

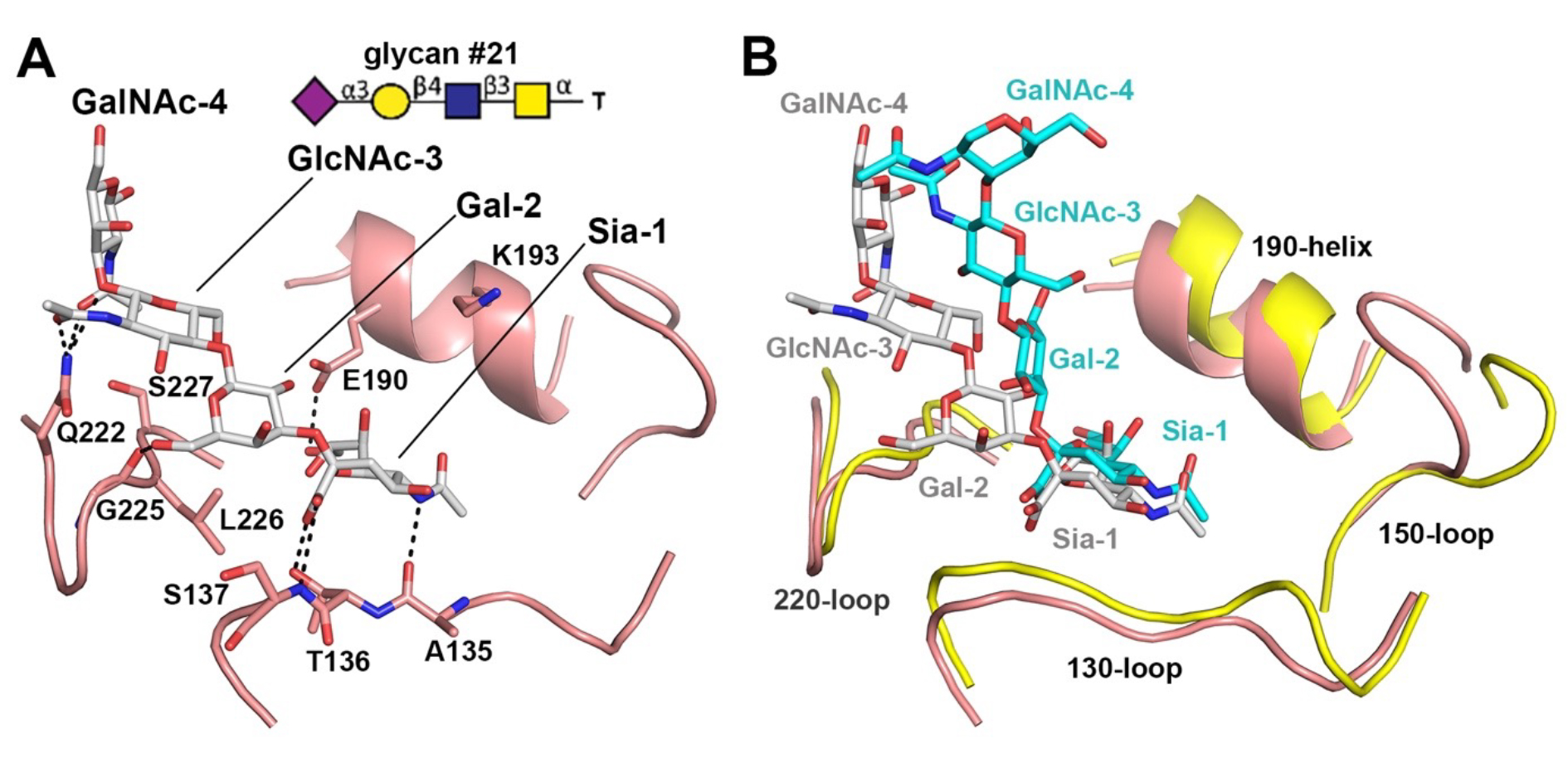

Supplement: S11 Fig — Avian O-linked glycan #21 (NeuAca2-3Galb1-4GlcNAcb1-3GalNAca-Thr) shares the same glycan structure with compound 25, but with Thr only in the peptide linker. (A) Glycan #21 bound to human H7 HA (PDB ID 4N63). (B) Superposition of human H7 HA with #21 compared to H5FR HA with compound 25. The H7 HA is in pink carbon atoms with ligand #21 in grey carbon atoms, and the H5FR HA in yellow carbon atoms with ligand 25 in cyan carbon atoms. (JPG) [file ppat.1013812.s015.jpg]

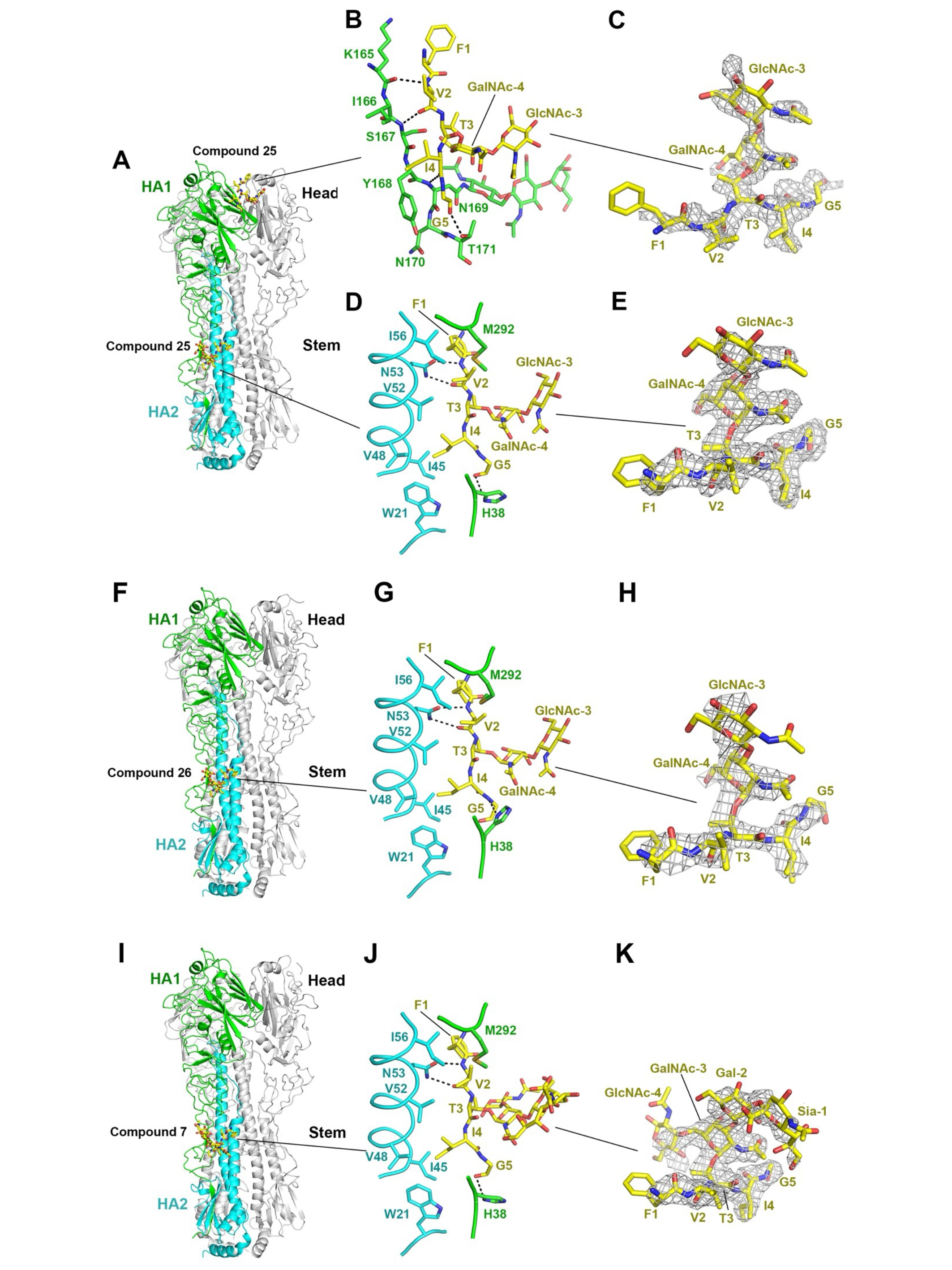

Supplement: S12 Fig — (A) to (E) H5FR H5 HA in complex with compound 25. In all of these figures, compound binding to the RBS has been removed for clarity. One HA protomer is colored with HA1 in green and HA2 in cyan and the other two protomers in grey. Compound 25 is colored in yellow carbon atoms, and the electron density (simulated annealing omit Fo-Fc map) for the glycopeptide is represented in a grey mesh and contoured at 2.5 σ. The same coloring scheme is used throughout this figure. (A) Overall structure of H5FR HA with 25. (B) Compound 25 also binds to a non-RBS site in the apex of the HA head domain. (C) Electron density map (as indicated above) for 25 in the HA head. (D) Compound 25 also binds to the HA stem domain. (E) Electron density map for 25 in the HA stem. (F) to (H) H5FR HA with compound 26 bound to the HA stem. (F) Overall structure of H5FR HA with 26. (G) Compound 26 interaction in the HA stem. (H) Electron density map for 26. (I) to (K) H5FR HA with compound 7 bound to the HA stem. (I) Overall structure of H5FR with 7. Compound 7 interacts with the HA stem. Electron density map for 7. (JPG) [file ppat.1013812.s013.jpg]

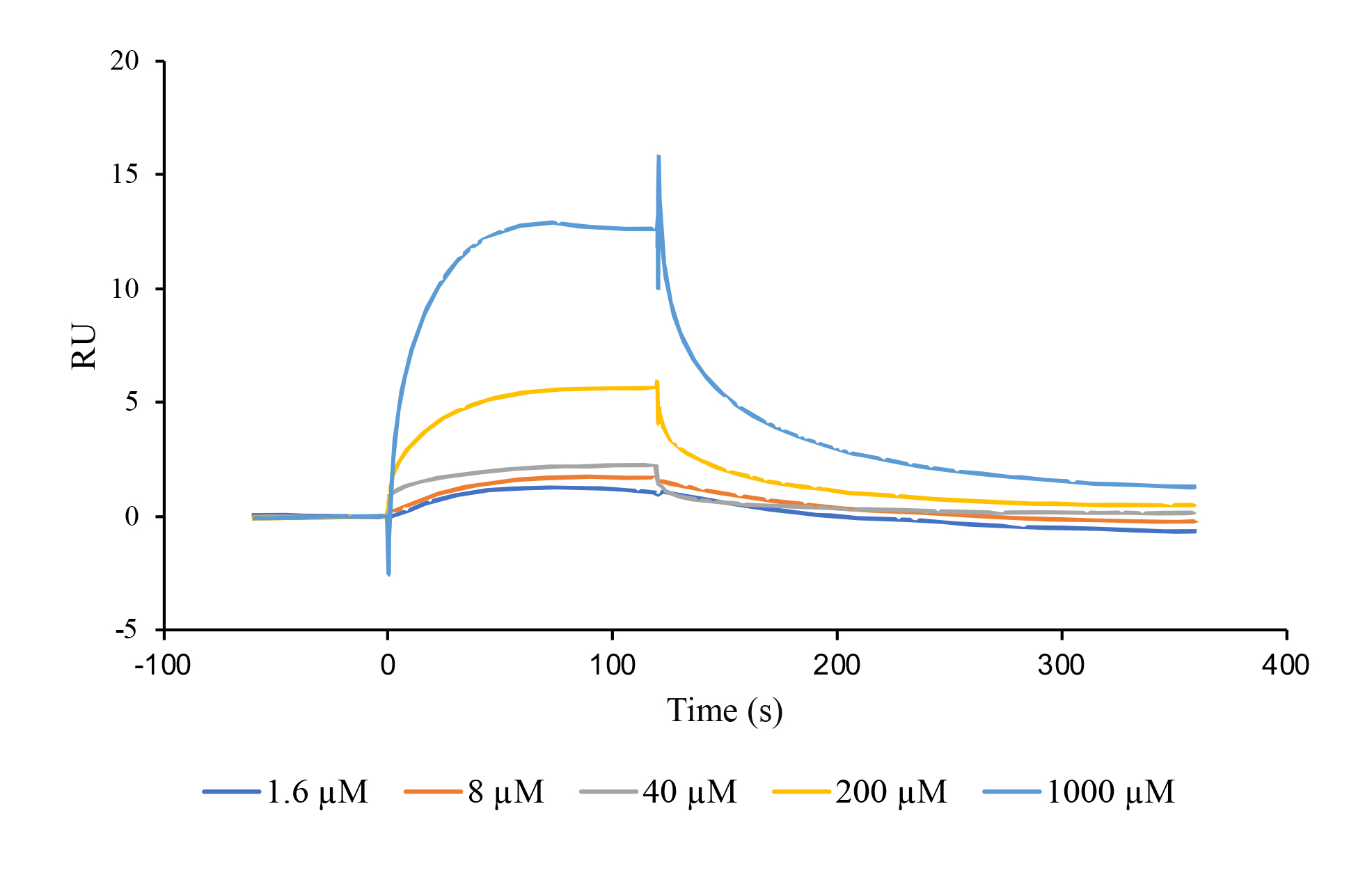

Supplement: S13 Fig — Signals (represented as response units, RU) were detected when serially diluted compound 2 solution was flowed through HA-immobilized sensor chips. Signals were recorded over time. Different colored lines represent different concentrations of 2, as indicated in the legend. KD value was estimated from steady-state affinity analysis. Since the upper end of the fitting curve did not saturate, we can only estimate the value of KD > 650 μM. (JPG) [file ppat.1013812.s014.jpg]
