# Supplementary material for: The receptor binding properties of H5Ny influenza A viruses have evolved to bind to avian-type mucin-like O-glycans
Source: PLoS Pathog. 2026 Jan 20;22(1):e1013812. doi: 10.1371/journal.ppat.1013812 (PMC12904578; doi:10.1371/journal.ppat.1013812)
Supplement: S1 Table — (DOCX) [file ppat.1013812.s002.docx]

**Table S1.** **Data collection and refinement statistics**

| Dataset | **H5FR HA**  ***apo*** | **H5FR HA**  **+ compound 26** | **H5FR HA**  **+ compound 25** |
| --- | --- | --- | --- |
| **Data Collection** |  |  |  |
| X-ray source | SSRL 12-1 | SSRL 12-1 | SSRL 12-1 |
| Wavelength (Å)  Space group | 0.97946  P2_1_2_1_2_1_ | 0.97946  P2_1_2_1_2_1_ | 0.97946  P2_1_2_1_2_1_ |
| Unit cell (Å) | *a* = 95.4,  *b* = 171.6,  *c* = 226.0 | *a* = 95.3  *b* = 171.9,  *c* = 225.6 | *a* = 95.2  *b* = 171.6,  *c* = 226.4 |
| Resolution (Å)^a^ | 45.56-1.94 (1.97-1.94) | 49.12-2.50 (2.54-2.50) | 43.87-1.98 (2.01-1.98) |
| Unique reflections^a^ | 270,800 (13,249) | 127,703 (6,285) | 254,864 (11,594) |
| Redundancy^a^ | 9.8 (6.7) | 7.4 (7.6) | 6.7 (5.5) |
| Average I/σ(I)^a^ | 20.9 (1.0) | 9.1 (0.9) | 16.1 (0.9) |
| Completeness (%)^a^ | 99.9 (99.1) | 99.9 (100) | 98.7 (90.5) |
| *R*_sym_^a,b^ | 0.14 (>1.0) | 0.20 (>1.0) | 0.12 (>1.0) |
| *R*_pim_^a,b^ | 0.05 (0.60) | 0.08 (0.70) | 0.05 (0.67) |
| CC_1/2_^a^ | 0.996 (0.538) | 0.985 (0.420) | 0.995 (0.459) |
| No. molecules per ASU^c^ | 3 | 3 | 3 |
|  |  |  |  |
| **Refinement** |  |  |  |
| Resolution (Å)^a^  Reflections in refinement | 45.56-1.94 (1.97-1.94)  270,603 | 49.12-2.50 (2.53-2.50)  127,534 | 43.87-1.98 (2.00-1.98)  254,641 |
| Refined residues | 1,476 | 1,497 | 1,496 |
| Refined waters | 1,810 | 665 | 1,793 |
| *R*_cryst_^a,d^ | 0.167 (0.329) | 0.174 (0.273) | 0.165 (0.308) |
| *R*_free_^a,e^ | 0.188 (0.358) | 0.208 (0.322) | 0.189 (0.313) |
| *B*-values (Å^2^)  Protein  Ligand  Water | 37  -  49 | 52  63  52 | 38  53  49 |
| Wilson *B*-values (Å^2^) | 30 | 42 | 32 |
| Ramachandran values (%)^f^ | 97.6, 0 | 96.4, 0.3 | 97.8, 0 |
| r.m.s.d. bond (Å) | 0.007 | 0.003 | 0.007 |
| r.m.s.d. angle (deg.) | 0.84 | 0.56 | 0.86 |
| PDB codes | 9NRR | 9NRS | 9NRT |

**Table S1.** **Data collection and refinement statistics - continued**

| Data set | **H5FR HA**  **+ compound 7** | **H5FR HA**  **+ LSTa** |
| --- | --- | --- |
| **Data Collection** |  |  |
| X-ray source | SSRL 12-1 | SSRL 12-1 |
| Wavelength (Å)  Space group | 0.97946  P2_1_2_1_2_1_ | 0.97946  P2_1_2_1_2_1_ |
| Unit cell (Å) | *a* = 95.7  *b* = 172.0,  *c* = 226.8 | *a* = 95.0  *b* = 177.0,  *c* = 224.6 |
| Resolution (Å)^a^ | 46.12-2.40 (2.44-2.40) | 48.97-2.90 (2.97-2.90) |
| Unique reflections^a^ | 148,008 (7,295) | 85,027 (5,581) |
| Redundancy^a^ | 8.5 (6.9) | 6.1 (6.1) |
| Average I/σ(I)^a^ | 17.1 (1.0) | 5.5 (0.7) |
| Completeness (%)^a^ | 99.9 (100) | 99.9 (99.9) |
| *R*_sym_^a,b^ | 0.16 (>1.0) | 0.27 (>1.0) |
| *R*_pim_^a,b^ | 0.06 (0.61) | 0.12 (0.80) |
| CC_1/2_^a^ | 0.990 (0.456) | 0.968 (0.402) |
| No. molecules per ASU^c^ | 3 | 3 |
|  |  |  |
| **Refinement** |  |  |
| Resolution (Å)^a^  Reflections in refinement | 46.12-2.40 (2.43-2.40)  147,849 | 48.97-2.90 (2.92-2.90)  84,890 |
| Refined residues | 1,495 | 1,482 |
| Refined waters | 1,090 | 183 |
| *R*_cryst_^a,d^ | 0.182 (0.293) | 0.178 (0.291) |
| *R*_free_^a,e^ | 0.206 (0.327) | 0.218 (0.321) |
| *B*-values (Å^2^)  Protein  Ligand  Water | 45  80  54 | 61  66  53 |
| Wilson *B*-values (Å^2^) | 41 | 53 |
| Ramachandran values (%)^f^ | 97.1, 0.2 | 94.1, 0.3 |
| r.m.s.d. bond (Å) | 0.003 | 0.009 |
| r.m.s.d. angle (deg.) | 0.54 | 1.02 |
| PDB codes | 9NRU | 9NRV |

^a^ Parentheses denote outer-shell statistics.

^b^ *R*_sym_ = ∑*_hkl_*∑*_i_* |*I_hkl,i_* - <*I_hkl_*>| /∑*_hkl_*∑*_i_* *I_hkl,i_* and *R*_pim_ = ∑*_hkl_*[1/(*N*-1)]^1/2^∑*_i_* |*I_hkl,i_* - <*I_hkl_*>| /∑*_hkl_*∑*_i_* *I_hkl,i_*, where *I_hkl,i_* is the scaled intensity of the i^th^ measurement of reflection *h*, *k*, *l*, < *I_hkl_*> is the average intensity for that reflection, and *N* is the redundancy. R_pim_ = Σ*_hkl_* (1/(n-1))^1/2^ Σ*_i_* | *I_hkl,i_* - *<I_hkl_>* | / Σ*_hkl_* Σ*_i_ I_hkl,i_*, where n is the redundancy

^c^ No. molecules for complexes refers to number of HA protomers per asymmetric unit (ASU), i.e. an HA trimer is in the ASU.

^d^ *R*_cryst_ = ∑*_hkl_* |*F_o_* - *F_c_*| / ∑*_hkl_* |*F_o_*|, where *F_o_* and *F_c_* are the observed and calculated structure factors.

^e^ *R*_free_ was calculated as for *R*_cryst_, but on 5% of data excluded before refinement.

^f^ The values are percentage of residues in the favored and outliers regions analyzed by MolProbity ^1^.
